# Severe Traumatic Brain Injury: A Case Report Challenging Traditional Prognostic Indicators

**DOI:** 10.7759/cureus.105927

**Published:** 2026-03-26

**Authors:** Tiffany Wood, Allison Lake, Joshua J Startup

**Affiliations:** 1 Physical Medicine and Rehabilitation, University of Michigan, Ann Arbor, USA

**Keywords:** neuropsychological testing, penetrating traumatic brain injury, post-traumatic amnesia, prolonged coma, severe combined traumatic brain injury, traumatic brain injury outcomes

## Abstract

This case report demonstrates the role of facility-based rehabilitation in the recovery of severe traumatic brain injury (TBI) and advocates for the pursuit of inpatient rehabilitation for this condition. This case describes a patient who suffered severe TBI, by all four initial prognostic criteria, and has had successful community reintegration because of a long facility-based course of therapy and a supportive community network. Methods include chart review and patient interview. This case report demonstrates that even in cases of severe TBI, where literature demonstrates a strong likelihood for poor outcome, there is the opportunity for substantial recovery.

## Introduction

Outcomes following severe traumatic brain injury (TBI) can range from full independence and societal reintegration to severe morbidity requiring full-time care. The pathophysiology of severe TBI is multifactorial, where an insult then initiates a series of changes within the brain. Insult directly causes a primary injury, which includes bleeding and skull defect and frequently leads to secondary injury that can include increased intracranial pressure, inflammation and oxidative stress, and hypoxia, which can develop over the following hours to months [1]. Prognosis after severe TBI is determined by evaluating three basic processes: developing conscious awareness, recovering higher-level cognitive functioning, and returning functional capacity [2]. TBI is classified via four criteria: the Glasgow Coma Scale (GCS), duration of loss of consciousness, duration of post-traumatic amnesia (PTA), and intracranial imaging findings. In 1994, Katz and Alexander used these criteria to assess patients suffering severe  TBI, following a total cohort of 243 patients over the course of a year, and found that there is a clear and predictable relationship between length of PTA and greater diffuse axonal injury (DAI), resulting in poorer outcomes [3]. Data over the past decade have revealed that even patients with persistent severe disability during acute hospitalization, of those who go to inpatient rehabilitation, 20% can progress to functional independence between one and 10 years post-injury [4]. There is variability of outcome based on the initial prognostic indicators, with patients experiencing severe TBI who can even return to sport [5]. Outcomes following TBI can be measured with standardized scales for functional, psychosocial/neurocognitive, and quality of life (QOL) metrics [6]. Severe TBI has shown a strong association with poor outcomes in different cognitive and functional domains. The Glasgow Outcome Scale was initially developed, then expanded via standardized approaches and principles for challenging assessments for borderline and difficult cases, but this still introduces some variability for future activities [7].  

In this case report, we review the prolonged recovery of a patient who suffered a severe TBI by all four criteria and yet has had a good recovery. We hope to champion the need for inpatient rehabilitation followed by continued follow-up and  outpatient support to promote successful societal reintegration and functional recovery after a severe TBI. 

## Case presentation

**Figure 1:**
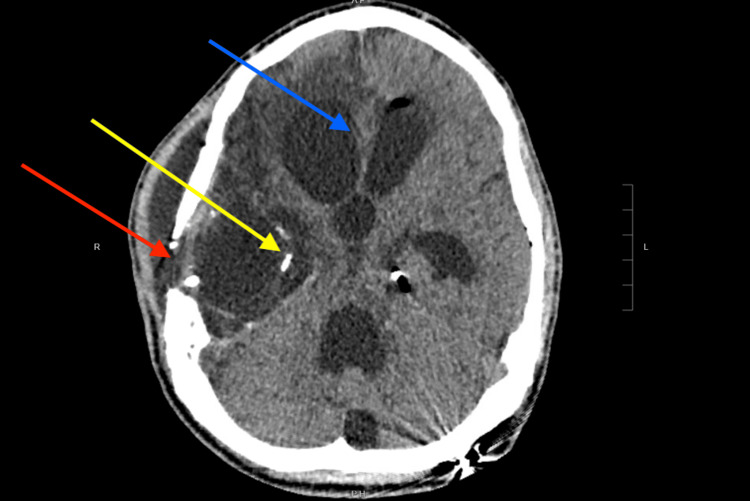
Initial non-contrast head CT Red arrow: open temporal bone defect. Yellow arrow: metallic bullet fragments. Blue arrow: subfalcine herniation.

A 20-year-old healthy male college student, studying environmental biology, suffered a gunshot wound in the setting of a public mass-shooting event. There was a direct insult to the right temporal bone. Resulting primary injuries included extensive intracranial and skull-base findings with right fronto-parietal subdural bleed, subarachnoid bleed originating from the circle of Willis with multiple right cranial fossa metallic bullet fragments, and subfalcine herniation (Figure 1).

His initial GCS was 3, and his best GCS within the first 24 hours after injury was 4-5. He had a prolonged hospital course significant for craniectomy, multiple skull base repairs, multiple infections, syndrome of inappropriate antidiuretic hormone (SIADH), and eventual ventriculoperitoneal (VP) shunt placement. He required stays at two inpatient rehabilitation facilities (IRF) due to his complications. He remained in PTA for greater than 12 weeks and in a coma for greater than three weeks, requiring supplemental feeding and tracheostomy. Following his second course of IRF, he was discharged home. He initially required assistance with activities of daily living (ADLs) such as bathing, eating, dressing, and mobility at home, then continued to progress in independence with intensive outpatient rehabilitation.  

**Figure 2:**
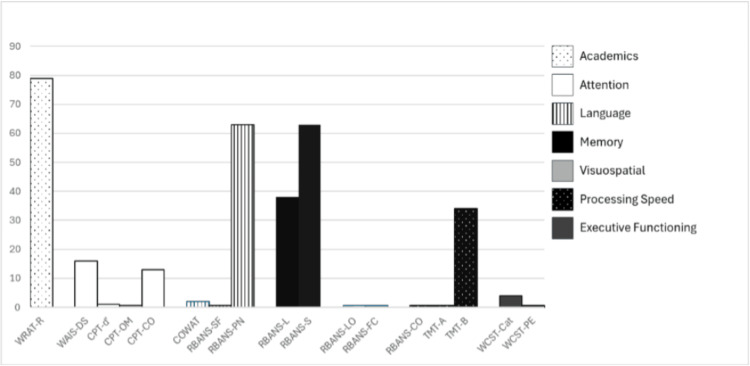
Percentile scores across domains of neuropsychological testing Tests included  Wide Range Achievement Test-5, Reading (WRAT-R) [8]; Wechsler Adult Intelligence Scale, Digit Span (WAIS-DS) [9]; Conners Continuous Performance Test (CPT) [10], detectability (d’), omissions (OM), and commissions (CO); Controlled Oral Word Association-Letter Fluency (COWAT) [11]; Repeatable Battery for the Assessment of Neuropsychological Status-Update (RBANS) [12], semantic fluency (SM), picture naming (PN), list recall (L), story recall (S), line orientation (LO), figure copy (FC), and coding (CO); Trail Making Test, oral (TMT-A, TMT-B) [13]; and Wisconsin Card Sorting Test (WCST) [14], categories (Cat) and perseverative errors (PE).

In consultation with Neuropsychology, approximately nine months post-injury, the patient acknowledged that his thinking had been “a little inhibited” since his injury and reported some difficulty counting. His mother and speech therapist described intact remote and recent memory but noted he did not recall any events surrounding his injury. He benefited from support for planning and self-monitoring. His cognitive endurance improved to up to one hour of focused work. Neuropsychological testing was completed, as reflected below (Figure 2).

For ADLs, the patient progressed to higher-level tasks like cooking and completing household chores as part of his in-home therapies. He could use his smartphone and easily recall passwords. He was managing multiple simultaneous text message chains but required occasional cuing to visually scan for an icon. He returned to previously enjoyed activities with his robust friend group, including playing darts, card games, board games, and bean bag toss. His mother stated that his skills in these areas have returned to his prior baseline. He returned to writing the newsletter for a college club by typing with his right hand and required assistance only in minor proofreading. His parents manage his medications, finances, and transportation. He was eating independently but required occasional cues for smaller bite sizes. His parents provided physical assistance in dressing, bathing, toilet transfers, and oral hygiene.

**Figure 3:**
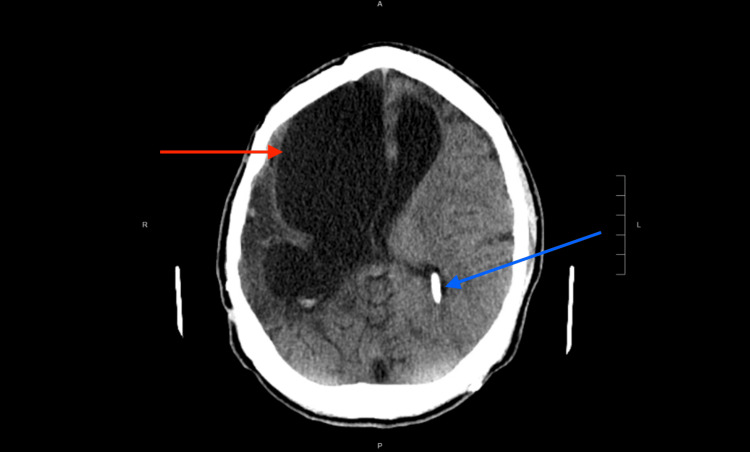
Recent non-contrast head CT Red arrow: cystic encephalomalacia in the right cerebral hemisphere. Blue arrow: left posterior approach ventriculostomy.

He continues to have residual left hemiparesis with spasticity and dystonia managed with a custom left ankle-foot orthosis and resting hand orthosis in conjunction with botulinum toxin injections, frequent stretching, and active home exercise programming. During the subsequent year, he further progressed with his independence in nearly all areas, eventually returning to college with accommodations including note-taking services, access to recordings, and increased time on testing and quizzes (Figure 3).

Concurrently with his physical activities, he was able to begin outdoor activities such as adaptive skiing with assistance from another person. He is on track for graduation from university. 

## Discussion

This case exemplifies the variability in TBI recovery and challenges the accuracy of early prognostic indicators. Specifically, this patient presented with a coma duration exceeding two weeks and PTA for greater than 12 weeks. Historically, such clinical outcomes predict a low probability for favorable recovery in the landmark studies by Katz and Alexander; no patients meeting these specific duration criteria achieved a “good recovery” on the Glasgow Outcome Scale (GOS) [3,15].   

Despite these indicators, this patient achieved a level of recovery that exceeds traditional clinical projections. While he might be classified as having "upper moderate disability" based on certain standardized assessments of future activities, his performance in prior vocational roles, specifically maintaining a 4.0 GPA as a full-time student at a top-tier university, aligns more accurately with a “lower good recovery” (GOSE 7) [16,17]. His discordance suggests that while PTA duration remains a sensitive index of DAI, it may not fully capture the recovery potential in cases of focal penetrating trauma or in patients with significant cognitive reserve [18]. 

Limitations of this case include that initial prognostic markers primarily predict outcomes correlated with DAI; in this patient’s case, his primary injury correlated with direct penetrating trauma as well as secondary infections.

## Conclusions

This case highlights that traditional prognostic markers like prolonged coma and PTA may not fully capture recovery potential, particularly in cases of focal penetrating trauma versus DAI. Despite meeting prognostic criteria to suggest permanent disability, this patient achieved a “good recovery,”  likely facilitated by his young age, high cognitive reserve, and access to intensive and nearly consecutive inpatient rehabilitation during the critical six-month recovery window. His continued recovery has been further supported by a robust familial network and accommodations in school, such as assistance with note-taking and increased time with assignments or examinations. His outcome underscores that despite early clinical indicators, some patients have the potential for significant recovery, cautioning against the withdrawal of services. In cases of severe TBI, consider individualized, multi-modal care to facilitate outcomes exceeding early expectations. 
